# Combining Hard Shell with Soft Core to Enhance Enzyme Activity and Resist External Disturbances

**DOI:** 10.1002/advs.202411196

**Published:** 2025-01-22

**Authors:** Yiwen Wang, Hongfei Tong, Shulan Ni, Kaiyuan Huo, Wenjie Liu, Xingjie Zan, Xiaodie Yuan, Shuangshuang Wang

**Affiliations:** ^1^ Department of Cardiology The First People's Hospital of Wenling Wenling Hospital of Wenzhou Medical University Wenling Zhejiang 317500 China; ^2^ Wenzhou Institute, University of Chinese Academy of Sciences Wenzhou Key Laboratory of Perioperative Medicine Wenzhou Zhejiang 325001 China; ^3^ Yongkang First People's Hospital of Wenzhou Medical University Jinhua Zhejiang China; ^4^ School of Materials Science and Engineering Zhengzhou University Zhengzhou 450001 China; ^5^ Key Laboratory of Precision Medicine for Atherosclerotic Diseases of Zhejiang Province Affiliated First Hospital of Ningbo University Ningbo Zhejiang 315010 China

**Keywords:** catalysis, core–shell structure, enzyme immobilization, hexahistidine metal assembly (HmA), zeolitic imidazolate framework (ZIF)

## Abstract

Immobilizing enzymes onto solid supports having enhanced catalytic activity and resistance to harsh external conditions is considered as a promising and critical method of broadening enzymatic applications in biosensing, biocatalysis, and biomedical devices; however, it is considerably hampered by limited strategies. Here, a core–shell strategy involving a soft‐core hexahistidine metal assembly (HmA) is innovatively developed and characterized with encapsulated enzymes (catalase (CAT), horseradish peroxidase, glucose oxidase (GOx), and cascade enzymes (CAT+GOx)) and hard porous shells (zeolitic imidazolate framework (ZIF), ZIF‐8, ZIF‐67, ZIF‐90, calcium carbonate, and hydroxyapatite). The enzyme‐friendly environment provided by the embedded HmA proves beneficial for enhanced catalytic activity, which is particularly effective in preserving fragile enzymes that will have been deactivated without the HmA core during the mineralization of porous shells. The enzyme encapsulated within a core–shell particle exhibits noteworthy resilience against harsh external conditions, including heat, organic solvents, and proteinase K. Additionally, no significant alteration in the catalytic behavior of the enzyme is observed after multiple cycles of usage. This study offers a novel approach for immobilizing enzymes and rendering them resistant to harsh external conditions, with potential applications in diverse fields, including biocatalysis, bioremediation, and biomedical engineering.

## Graphical abstract

A core‐shell strategy is innovatively developed, consisting of soft core of hexahistidine metal assembly (HmA) with encapsulated enzymes (CAT, HRP, and GOx) and hard porous shells (ZIF‐8, ZIF‐67, ZIF‐90, calcium carbonate and hydroxyapatite). Enzyme@HmA@shell maintaine or enhance the catalytic activity of encapsulated enzyme and protected enzymes from external disturbs, such as heat, organic solvents, and proteases.

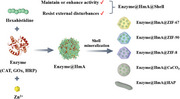

## 1 Introduction

Enzymes are a class of biocatalytic proteins involved in most biochemical reactions that occur in living systems and are considered to be one of the most important biological macromolecules required for life‐sustaining biotransformation. Enzymes are now widely used in biosensing,^[^ ^1^, ^2^ ^]^ biocatalysis,^[^ ^3^, ^4^, ^5^ ^]^ and biomedical devices^[^ ^6^ ^]^ because of their favorable catalytic properties, such as efficient regio‐ and stereo‐specific reactions, to precisely generate desired molecules. The unique three‐dimensional structure formed during long‐term evolution is the key to the excellent biocatalytic functions of enzymes. However, this unique 3D structure is susceptible to external perturbations (such as pH or temperature changes and organic solvent treatments) and may cause the deactivation of an enzyme; this considerably hinders the industrial application and outstanding performance of enzyme‐related devices.

Enzymes have been immobilized on solid supports (such as inorganic particles, polymer fibers, and soft hydrogels) to facilitate the recycling of enzyme catalysts in numerous studies.^[^ ^7^, ^8^, ^9^ ^]^ Maintaining enzyme activity during the immobilization process and preventing external interference after immobilization are critical in the field of enzyme immobilization. Mandal et al. successfully immobilized enzymes onto a diazo‐activated silica gel via multipoint covalent bonding with tyrosine residues. This approach significantly enhanced the catalytic stability of the enzymes and allowed the enzymes to maintain their activity across a broad pH range in the presence of organic solvents and under prolonged exposure to room‐temperature conditions.^[^ ^10^, ^11^, ^12^, ^13^, ^14^ ^]^ Utilizing porous materials is widely acknowledged as an optimal approach for enzyme immobilization owing to their large surface areas, which enable enhanced enzyme immobilization and the transport of enzyme‐catalyzed media.^[^ ^15^, ^16^ ^]^ Since Liu et al. conducted their pioneering work,^[^ ^17^ ^]^ metal organic frameworks (MOFs) constructed by coordinating metal ions or ion clusters with organic ligands have become one of the most popular host materials for fixing enzymes because of their structural diversity, porosity, large specific surface area, and tunable pore structure. Furthermore, the stability of MOFs under heat and in organic solvents is a prerequisite for enzymes to resist external perturbations.

Various strategies for enzyme fixation have been developed based on MOF matrices and can be broadly classified into two categories: immobilization on the outer surface of MOFs through post‐modification reactions (chemical bonding or physical adsorption)^[^ ^18^, ^19^ ^]^ and in situ encapsulation into the MOFs via mineralization.^[^ ^20^, ^21^ ^]^ For post‐modification reactions, appropriately regulating the interactions between the enzyme and MOF to avoid enzyme inactivation remains challenging. Moreover, most enzymes immobilized on a support surface become vulnerable to external environmental interference.^[^ ^22^, ^23^ ^]^ Fixing enzymes within the pores of MOFs seems promising against external disturbances, but the limited pore size (< 2 nm) of MOFs inhibits most enzymes from entering the pores via post‐modification reactions.^[^ ^24^ ^]^ For in situ encapsulation, the harsh synthesis conditions required for MOFs and the hydrophobic microenvironment in the pores of MOFs are fatal to enzyme activities.^[^ ^25^, ^26^ ^]^ In addition, the catalytic performance of encapsulated enzymes is significantly compromised by the sluggish mass transport capacity inside MOFs. Many studies have been conducted to overcome the shortcomings of MOFs. Cheng et al. showed that altering the hydrophilicity and ratio of linkers fine‐tuned the microenvironment of the MOF and optimized the activities of the enzyme trapped inside.^[^ ^26^ ^]^ Ouyang et al. reported a strategy for encapsulating a wide range of enzymes into MOFs by pre‐nucleating cystine‐rich amino acid clusters around the encapsulated enzymes.^[^ ^21^ ^]^ Lou et al. proposed a competitive coordination strategy for tailoring the bioactivities of enzymes trapped in MOFs using an amorphous zinc nucleotide gel as a template.^[^ ^27^ ^]^

Core–shell structures are ubiquitous in biological systems at multiple scales, including cells, fish eggs, and embryos. Particles having core–shell structures, consisting of multiple compartmentalized regions in which the outer shell encloses one or more cores, display unique performances because of the advantages associated with the designed core, shell material, geometry, etc.^[^ ^28^, ^29^, ^30^, ^31^, ^32^ ^]^ In a core–shell structure, the core provides a relatively closed environment that is beneficial for the activities of the encapsulated components. The shell plays a role in regulating mass transfer from the outer environment to the inner core, isolating packaged components from the surrounding environment, and protecting the packaged components from external turbulence.^[^ ^33^ ^]^ The structure and material of the shell or core can be independently designed and adjusted; thus, hierarchical architectures can be fabricated.^[^ ^34^, ^35^ ^]^ Although extensive research on the core–shell structure in various disciplines has been conducted,^[^ ^36^, ^37^ ^]^ to our knowledge, this structure has not been applied to enzyme fixation and resistance to external perturbations.

**Scheme 1:**
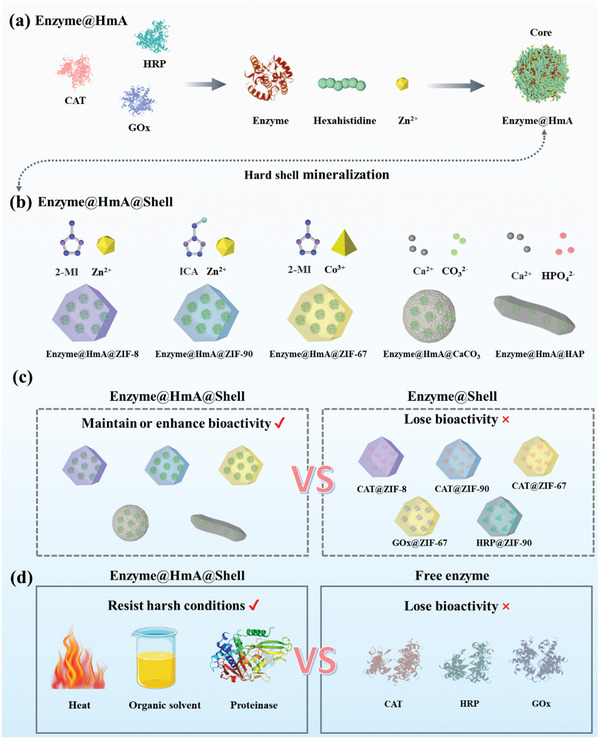
Schematic illustration of a) generating enzyme@HmA@shell by adding Zn^2+^ into a mixture of hexahistidine and an enzyme at a pH of 8.5; b) producing enzyme@HmA@shell by mineralizing various hard shells onto enzyme@HmA; c) the capacity of encapsulated enzymes to maintain or enhance bioactivity; and d) the capacity of encapsulated enzymes to resist external environmental disturbances (heat, organic solvents, and proteinase).

In our previous study, a library of proteins having different molecular weights and isoelectronic points was easily encapsulated in situ into a hexahistidine metal assembly (HmA) by employing an enzyme‐friendly process, in which the bioactivity of the protein could be stored.^[^ ^38^, ^39^, ^40^ ^]^ In this study, we developed a core–shell structure consisting of a soft core of a HmA and various hard shells, including three zeolite imidazolate frameworks (ZIFs, a subclass of MOFs), namely, ZIF‐8, ZIF‐67, and ZIF‐90), and two inorganic particles (calcium carbonate (CaCO_3_) and hydroxyapatite (HAP)), for enzyme fixation and resistance to external disturbances. The core–shell structure development was divided into two steps: 1) encapsulation of the enzyme into the HmA to generate the soft core, enzyme@HmA (**Scheme** **1**a), and 2) mineralization of the hard shells onto enzyme@HmA to generate enzyme@HmA@shell (Scheme 1b). Our data revealed that this strategy can be applied to various enzymes (catalase (CAT), horseradish peroxidase (HRP), glucose oxidase (GOx), and cascade enzymes (GOx+CAT)) while maintaining or enhancing the bioactivity of the encapsulated enzyme (Scheme 1c) and resisting external environmental disturbances (heat, organic solvents, and proteinase) (Scheme 1d). Importantly, enzyme@HmA@shell preserved the biological activity of the encapsulated enzyme after dozens of catalytic cycles. We believe that this strategy will significantly promote the development of enzyme immobilization and enzyme‐related devices and expand the applications of enzymes.

## 2 Results and Discussion

Numerous naturally occurring enzymes have been discovered, and three of the most extensively investigated enzymes (GOx, HRP, and CAT) were selected for this study. The enzymes (GOx, HRP, and CAT) were embedded in an HmA by using previously reported methods.^[^ ^38^, ^40^, ^41^, ^42^ ^]^ The HmA was used as the core owing to its ability to efficiently encapsulate proteins and reserve the bioactivities of encapsulated proteins.^[^ ^38^, ^40^, ^41^ ^]^ Five porous materials, namely, three commonly used ZIFs for enzyme immobilization (ZIF‐8, ZIF‐90, and ZIF‐67) and two widely studied inorganic particles (CaCO_3_ and HAP), were selected as shells to demonstrate the advantages and versatility of the core–shell strategy. The encapsulation process involved two sequential steps: (1) Zn^2+^ was added dropwise to the mixed solution of the enzyme and His_6_ at room temperature to generate the core material, enzyme@HmA (Scheme 1a); (2) enzyme@HmA was then introduced into various ligand solutions of the shell materials, and metal ions were added to mineralize the hard shell onto the soft core and generate enzyme@HmA@shell (Scheme 1b). As listed in Table S1 (Supporting Information), the sizes of all enzyme@HmA samples are in the range of 180–220 nm, as observed in the scanning electron microscopy (SEM) image of enzyme@HmA (Figure S1, Supporting Information). The polydispersity indices of the enzyme@HmA particles are in the range of 0.1–0.25 (Table S1, Supporting Information), indicating that the particles are dispersed well. After the mineralization of the enzyme or enzyme@HmA with the shell material, samples (enzyme@shell (without the HmA core) and enzyme@HmA@shell) of several microns in size are produced (Table S1, Supporting Information). CAT is used as an example enzyme to illustrate the success of the core–shell strategy. SEM observations reveal that the morphology of CAT@HmA@shell is almost identical to that of CAT@shell (Figure S2, Supporting Information). As reported in previous studies, CAT@HmA@ZIF‐8/90/67 have a dodecahedral structure^[^ ^43^, ^44^ ^]^ and CAT@HmA@CaCO_3_ and CAT@HmA@HAP are spherical and needlelike,^[^ ^45^, ^46^ ^]^ respectively (Figure S2, Supporting Information). Compared with CAT@shell, the surface of CAT@HmA@shell appears coarser and exhibits protruding particles (Figure S2, Supporting Information). Furthermore, the X‐ray diffraction patterns of CAT@shell and CAT@HmA@shell exhibit a high degree of similarity to the previously reported patterns^[^ ^47^, ^48^, ^49^, ^50^ ^]^ of the corresponding shell (Figure S3, Supporting Information). The encapsulation efficiency (EE%) of these enzymes in the HmA and the resulting HmA@shell is tested using high‐performance liquid chromatography and ultraviolet–visible spectroscopy, respectively. As listed in Table S1 (Supporting Information), the EE% of CAT in the HmA and HmA@shell is approximately 98% and 88–93%, respectively, depending on the shell material. The locations of CAT and the HmA core in the mineralized shell particles are visualized by applying confocal laser scanning microscopy (CLSM) after labeling CAT and hexahistidine with rhodamine B (red) and fluorescein isothiocyanate (green), respectively. As displayed in Figure S4 (Supporting Information), the green and red colors are uniformly distributed within the CAT@HmA@shell particles. A high degree of overlap between the green and red colors is observed within the CAT@HmA@shell particles, confirming that the enzyme combined with the HmA is embedded in the enzyme@HmA@shell particles. Under thermal treatment (300 °C, 1 h) (Figure S5, Supporting Information), ZIF‐8 does not display any pores, whereas both CAT@ZIF‐8 and CAT@HmA@ZIF‐8 exhibit structural collapse, further indicating the presence of CAT and CAT@HmA inside ZIF‐8.^[^ ^17^ ^]^ These findings suggest that enzyme@HmA, which serves as the core, is evenly incorporated into the mineralized particles and that the introduction of enzyme@HmA does not alter the crystal structure of the mineralized shell layer.

**Figure 1:**
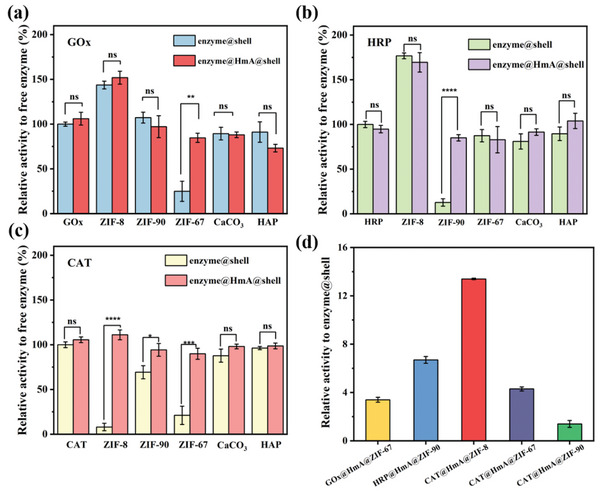
Catalytic activity of enzyme@shell and enzyme@HmA@shell relative to that of the corresponding free enzyme: a) GOx, b) HRP, and c) CAT. d) Activity of enzyme@HmA@shell relative to that of the corresponding enzyme@shell, which is manufactured by directly mineralizing the enzyme within the shell material. enzyme@HmA@shell is created by encapsulating the enzyme in the HmA and subsequently mineralizing the shell material. Shells include ZIF‐8, ZIF‐90, ZIF‐67, CaCO_3_, and HAP. Data are presented as the mean ± standard deviation. *p* values are determined by conducting t‐tests (^ns^p > 0.05, *p ≤ 0.05, **p ≤ 0.01, ***p ≤ 0.001, ****p ≤ 0.0001).

After the enzymes are encapsulated into the HmA, the enzymatic activity of enzyme@HmA becomes comparable to that of free enzymes (GOx, HRP, and CAT) (Table S2, Supporting Information). This suggests that the HmA preserves the activities of the encapsulated enzymes, consistent with previous studies.^[^ ^38^, ^40^ ^]^ The catalytic activities of the enzymes (GOx, HRP, and CAT) immobilized via direct mineralization (enzyme@shell) or the core–shell strategy (enzyme@HmA@shell) were further tested. We normalized the catalytic activities of free enzymes at room temperature. The catalytic activities of all the enzymes, enzyme@shell, enzyme@HmA, and enzyme@HmA@shell, were examined relative to those of the free enzyme at room temperature. The activity after being normalized relative to the activity of the corresponding free enzymes is shown in **Figure** **1**a–c. In general, for enzymes that do not exhibit a remarkable reduction in activity during direct mineralization of the shell layer, no significant difference in enzyme activity is observed between enzyme@shell and enzyme@HmA@shell. However, when direct mineralization of the shell layer results in a decline in enzyme activity (such as in GOx@ZIF‐67, HRP@ZIF‐90, CAT@ZIF‐8, CAT@ZIF‐67, and CAT@ZIF‐90), the core–shell approach markedly enhances the enzyme activity. Specifically, for GOx (Figure 1a), the relative activities of GOx@ZIF‐67 and GOx@HmA@ZIF‐67 are found to be 24.9 ± 11.3% and 84.7 ± 5.2%, respectively. In the case of HRP (Figure 1b), the activities of HRP@ZIF‐90 and HRP@HmA@ZIF‐90 are reduced to 12.7 ± 4.2% and increased to 85.1 ± 3.6%, respectively. In the case of CAT (Figure 1c), the activities of CAT@ZIF‐8, CAT@ZIF‐67, and CAT@ZIF‐90 are 8.1 ± 4.2%, 21.1 ± 10.3%, and 69.3 ± 7.3%, respectively. After the implementation of the core–shell strategy, the relative activities of CAT@HmA@ZIF‐8, CAT@HmA@ZIF‐67, and CAT@HmA@ZIF‐90 increase to 111.1 ± 5.5%, 89.9 ± 6.2%, and 94.3 ± 7.2%, respectively. To more clearly demonstrate the benefits of the core–shell strategy, for enzymes with markedly reduced activity in enzyme@shell, the enzyme activity in enzyme@HmA@shell is normalized relative to that in enzyme@shell. As shown in Figure 1d, for GOx@ZIF‐67, HRP@ZIF‐90, CAT@ZIF‐8, CAT@ZIF‐67, and CAT@ZIF‐90, the activities of the corresponding core–shell systems increase 3.4‐, 6.7‐, 13.7‐, 4.3‐, and 1.4‐fold, respectively. These data reveal that the HmA core maintains enzymatic activities during mineralization.

To increase the resistance to organic solvents, the catalytic activities of the free enzymes, enzyme@HmA, enzyme@shell, and enzyme@HmA@shell were evaluated after incubation with three typical organic reagents having different polarities (dimethyl sulfoxide (DMSO), MeOH, and CH_2_Cl_2_) at room temperature for 10 min. As listed in **Table** **1**, after the treatment with organic solvents, the catalytic activities of all the tested samples decline. However, the extent of this decline varies considerably among the individual systems. The free enzymes demonstrate near‐total deactivation, whereas enzyme@HmA exhibits a significant decrease in catalytic activity, ranging from 3% to 32% across various types of enzymes and organic solvents. The catalytic activities of enzyme@shell and enzyme@HmA@shell slightly decline when protected by a ZIF shell. In contrast, the CaCO_3_ and HAP shells demonstrate no protective effect on enzyme activities, and this may be attributed to their large pore sizes.^[^ ^51^ ^]^ These results indicate that the core–ZIF strategy retains the catalytic activity of the enzyme and offers a protective effect in organic solvents.

**Table 1:** Relative activities of free enzymes, enzyme@HmA (core), enzyme@shell (enzyme directly immobilized on the shell), and enzyme@HmA@shell (enzyme immobilized via core–shell strategy) after treatment with different polar organic solvents for 10 min at room temperature. The enzymes are GOx, HRP, and CAT; the shells are ZIF‐8, ZIF‐67, ZIF‐90, CaCO_3_, and HAP. The data are normalized against the activity of free enzymes at room temperature.

Types of particles	GOx			HRP			CAT		
	MeOH	DMSO	CH_2_Cl_2_	MeOH	DMSO	CH_2_Cl_2_	MeOH	DMSO	CH_2_Cl_2_
Free enzyme	6.4±2.4	7.1±6.8	9.2±2.4	12.6±7.5	8.1±4.4	10.8±4.0	2.2±0.9	7.4±2.1	9.4±5.9
Enzyme@HmA	31.2±13.2	24.9±5.2	17.5±9.0	17.2±9.7	13.7±4.8	19.7±5.9	6.9±4.3	7.4±1.5	3.4±1.5
Enzyme@ZIF‐8	137.7±5.1	129.8±3.8	107.9±6.7	187.9±8.3	194.5±8.8	134.8±9.4	2.1±1.3	6.9±2.6	6.9±0.7
Enzyme@HmA@ZIF‐8	129.7±7.5	134.7±2.0	122.5±13.3	167.0±2.6	172.0±4.3	159.0±17.0	103.0±6.5	100.1±7.7	94.5±4.7
Enzyme@ZIF‐67	14.7±8.5	21.9±3.0	14.8±6.5	84.9±13.6	91.7±9.5	61.8±2.9	13.7±4.8	19.9±6.9	16.8±9.7
Enzyme@HmA@ZIF‐67	56.7±8.1	53.8±5.6	49.7±9.0	86.7±11.2	74.8±7.1	69.7±8.0	86.7±7.3	73.8±6.4	79.7±7.9
Enzyme@ZIF‐90	89.9±5.6	92.7±8.2	81.8±11.0	10.8±1.1	16.7±3.0	13.4±6.4	49.9±8.6	52.7±3.8	41.8±2.4
Enzyme@HmA@ZIF‐90	91.5±2.7	84.2±6.7	83.1±10.3	75.3±5.0	81.7±8.5	79.4±8.0	91.5±2.1	84.2±4.9	83.1±2.3
Enzyme@CaCO_3_	8.1±6.9	6.4±1.9	13.7±2.5	8.1±6.9	12±7.0	10.4±1.2	8.1±1.1	6.1±2.9	13.7±2.2
Eenzyme@HmA@CaCO_3_	8.4±5.1	9.1±2.7	4.5±1.1	16.7±8.4	21.7±4.2	10.7±8.7	10.9±6.7	8.7±1.6	6.6±2.8
Enzyme@HAP	13.6±9.5	7.8±3.8	3.9±2.9	13.6±9.5	9.4±1.6	4.5±2.9	13.6±1.7	7.8±2.7	3.8±2.8
Enzyme@HmA@HAP	10.6±2.6	10.7±2.1	8.1±6.8	11.9±9.1	9.7±4.1	6.4±4.2	8.8±1.1	12.4±5.8	9.7±3.6

Compared with GOx and HRP (Figure 1a–d), CAT is more susceptible to inactivation in the tested ZIFs, probably because of its inherent sensitivity to the microenvironment of the ZIFs.^[^ ^25^, ^26^ ^]^ In the case of CAT with the ZIF‐8 shell, the activity of CAT@HmA@ZIF‐8 is 13.7 times higher than that of CAT@ZIF‐8. The substantial enhancement in catalytic efficacy leads to the selection of CAT and ZIF‐8 as the model system for subsequent studies with the aim of elucidating the influence of the core–shell strategy on the protection and activity retention of enzymes.

**Figure 2:**
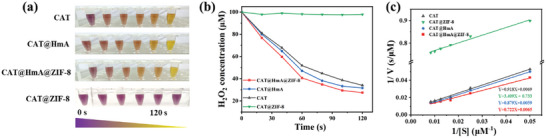
a) Optical images of color changes in FOX solutions treated with different samples (CAT, CAT@HmA, CAT@ZIF‐8, and CAT@HmA@ZIF‐8) and mixed with H_2_O_2_ solution within 120 s. b) Decomposition curves of H_2_O_2_ treated with different samples with respect to the reaction time. c) Lineweaver–Burk plots for determining the kinetic parameters of CAT, CAT@HmA, CAT@ZIF‐8, and CAT@HmA@ZIF‐8.

The catalytic activities of free CAT, CAT@HmA, CAT@ZIF‐8, and CAT@HmA@ZIF‐8 were investigated by using H_2_O_2_ as the substrate and ferrous‐oxidized xylenol orange (FOX) as a colorimetric indicator.^[^ ^52^ ^]^ **Figure** **2**a depicts that the CAT@ZIF‐8 group maintains a purple color, with no change observed after the 120‐s time frame. In contrast, the color of the FOX solutions treated with CAT, CAT@HmA, and CAT@HmA@ZIF‐8 changes from purple to yellow. A notable finding is presented in Figure 2b: H_2_O_2_ for CAT@ZIF‐8 is not consumed at all, and the concentration of H_2_O_2_ decreases with the reaction time for the other three samples. These results indicate that CAT loses its catalytic activity in CAT@ZIF‐8 and that the presence of the HmA in the core is beneficial for retaining the catalytic activity of the encapsulated CAT. Kinetic parameter measurements for CAT, CAT@HmA, CAT@HmA@ZIF‐8, and CAT@ZIF‐8 are conducted by utilizing Lineweaver–Burk plots (Figure 2c). The V_max_ values indicate the maximum rate of the enzymatic reaction at equilibrium, corresponding to the reciprocal of the y‐intercept in the Lineweaver–Burk plot. Km represents the Michaelis constant, which reflects the binding affinity between the enzyme and substrate. This constant is determined from the negative reciprocal of the x‐intercept in the Lineweaver–Burk plot. A smaller K_m_ value indicates a stronger binding ability between the enzyme and substrate.^[^ ^53^ ^]^ The kinetic parameters are 144.9, 169.4, 153.8, and 1.4 µM/s for CAT, CAT@HmA, CAT@HmA@ZIF‐8, and CAT@ZIF‐8, respectively, whereas the corresponding Km values are 133.0, 148.9, 111.0, and 4.6 µM (Table S3, Supporting Information). Surprisingly, CAT@ZIF‐8 exhibits a significantly reduced V_max_ (1.4 µM s^−1^) compared with other samples. Additionally, CAT@HmA@ZIF‐8 shows a lower Km and higher V_max_ than native CAT. These data imply that the enhanced catalytic activity observed for certain immobilized enzymes may be correlated with improved binding capacity with the substrate.^[^ ^54^ ^]^

**Figure 3:**
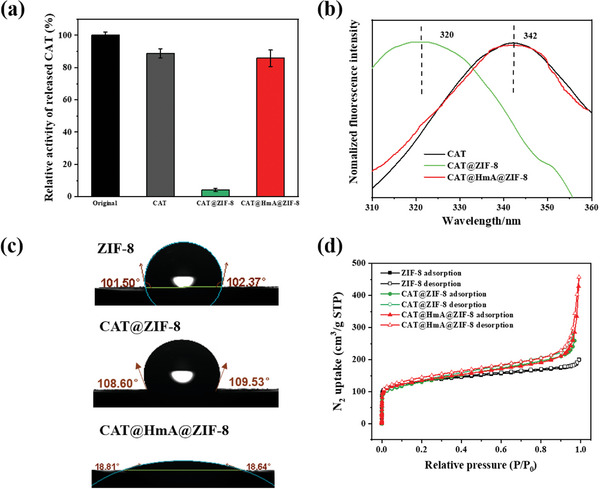
a) Catalytic activities of CAT, CAT@ZIF‐8, and CAT@HmA@ZIF‐8 after acid hydrolysis are compared with that of CAT without acid treatment. b) Fluorescence spectroscopy profiles of CAT, CAT@ZIF‐8, and CAT@HmA@ZIF‐8. c) Water contact angles of ZIF‐8, CAT@ZIF‐8, and CAT@HmA@ZIF‐8. d) Nitrogen adsorption–desorption isotherms of ZIF‐8, CAT@ZIF‐8, and CAT@HmA@ZIF‐8.

CAT in CAT@HmA, CAT@ZIF‐8, and CAT@HmA@ZIF‐8 is released at a low pH by disassembling the HmA and ZIF‐8.^[^ ^39^, ^55^ ^]^ A pH of 5.0 is selected to release CAT from CAT@HmA, CAT@ZIF‐8, and CAT@HmA@ZIF‐8, because the catalytic activity of CAT is retained at this pH value (Figure S6, Supporting Information). The catalytic activity of CAT released from CAT@HmA and CAT@HmA@ZIF‐8 is comparable to that of native CAT (**Figure** **3**a). By contrast, only 4.1% activity is retained in CAT released from CAT@ZIF‐8 (Figure 3a), suggesting that CAT is deactivated during the mineralization process for ZIF‐8. In the Fourier transform infrared (FTIR) spectroscopy analysis (Figure S7, Supporting Information), both CAT@ZIF‐8 and CAT@HmA@ZIF‐8 exhibit the characteristic amide I vibration bands of CAT in the wavelength range of 1610–1700 cm^−1^.^[^ ^25^ ^]^ The peak position of CAT in CAT@HmA@ZIF‐8 at 1651 cm^−1^ is akin to that of native CAT, implying that the protein structure is preserved in CAT@HmA@ZIF‐8. By contrast, the amide I vibration peak of CAT@ZIF‐8 shows a pronounced red shift to 1672 cm^−1^ (Figure S7, Supporting Information), indicative of the deactivation of CAT in CAT@ZIF‐8, which is attributed to the hydrophobic interactions between CAT and ZIF‐8.^[^ ^25^ ^]^ The λ_max_ of CAT at 342 nm originates from the exposure of tryptophan residues and is very sensitive to the surrounding environment.^[^ ^56^, ^57^ ^]^ Fluorescence spectroscopy (Figure 3b) reveals that compared with native CAT, the λ_max_ of CAT in ZIF‐8 undergoes a blue shift from 342 to 320 nm upon encapsulation. No such shift is observed for CAT encapsulated in HmA@ZIF‐8, suggesting that the HmA mitigates the conformational changes induced by ZIF‐8.^[^ ^56^ ^]^ Figure S8 (Supporting Information) displays that the negative band of native CAT at 206 nm undergoes a blue shift to 204 nm upon encapsulation in ZIF‐8 (CAT@ZIF‐8), whereas no significant band shift is observed for CAT@HmA@ZIF‐8. This result indicates a structural alteration of CAT within ZIF‐8 and is consistent with the FTIR and fluorescence spectroscopy results mentioned previously.^[^ ^58^ ^]^ The water contact angle of CAT@HmA@ZIF‐8 is markedly lower than those of ZIF‐8 and CAT@ZIF‐8 (Figure 3c), suggesting that the embedded HmA modifies the microenvironment of CAT within ZIF‐8. The N_2_ adsorption–desorption isotherms of both ZIF‐8 is classified as type I, whereas that of CAT@ZIF‐8 and CAT@HmA@ZIF‐8 are of type II (Figure 3d), indicating the presence of a hierarchical porous structure in CAT@ZIF‐8 and CAT@HmA@ZIF‐8.^[^ ^59^ ^]^ The pore sizes are estimated from the isotherm curves. As presented in Figure S9 and Table S4 (Supporting Information), the pore size and surface area of CAT@ZIF‐8 are very close to those of CAT@HmA@ZIF‐8. This suggests that the differences in catalytic activity between CAT@ZIF‐8 and CAT@HmA@ZIF‐8 cannot be attributed to variations in pore structure.

**Figure 4:**
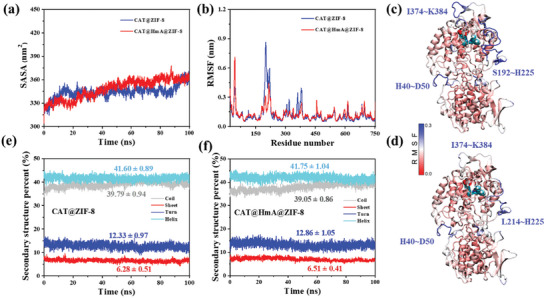
a) SASAs of CAT in CAT@ZIF‐8 and CAT@HmA@ZIF‐8 with respect to the simulation time. b) RMSF distribution of CAT protein in the CAT@ZIF‐8 and CAT@HmA@ZIF‐8 systems. Distribution of the corresponding regions with large changes in the protein structure in c) CAT@ZIF‐8 and d) CAT@HmA@ZIF‐8. Changes in CAT secondary structure content in e) CAT@ZIF‐8 and f) CAT@HmA@ZIF‐8 with respect to the simulation time.

To further elucidate the differences in the activities of CAT in the presence and absence of His_6_ in ZIF‐8, molecular dynamics (MD) simulations were performed to investigate the effects on the structural dynamics of the protein. The solvent‐accessible surface areas (SASA) of CAT@ZIF‐8 and CAT@HmA@ZIF‐8 during the MD simulations are presented in **Figure** **4**a. The SASA values for both systems display notable fluctuations within the first 20 ns of the MD simulations, likely attributable to the increased influence of the surrounding molecules on the CAT protein during this early stage; the increased influence results in greater structural variability and subsequently affects the SASA measurements. After 60 ns, the SASA of CAT@HmA@ZIF‐8 stabilizes at an average of 359.55±4.33 nm^2^. By contrast, the SASA of CAT@ZIF‐8 initially decreases slightly and then increases pronouncedly after 90 ns; specifically, it increases from 346.70±3.94 nm^2^ during the interval from 60 to 80 ns to 358.14±5.64 nm^2^ during the interval from 90 and 100 ns, suggesting that structural alterations in the CAT protein are more significant within CAT@ZIF‐8. Root‐mean‐square fluctuation (RMSF) is a quantitative measure used to assess the flexibility of amino acid residues in proteins. The simulations reveal that the amino‐acid‐flexibility distribution trend of CAT is consistent between the two systems (Figure 4b). More specifically, a comparison between Figure 4c,d reveals that the distribution of highly flexible regions in CAT is largely consistent in both systems (H40‐D50, S192‐H225, and I374‐K384). When His_6_ is added, the amino acid flexibility of CAT is significantly reduced in certain regions, suggesting that His_6_ contributes to increased structural stability. To gain deeper insight into the structural changes induced by His_6_, alterations in the secondary structure of CAT were examined. As depicted in Figure 4e,f, the incorporation of His_6_ results in a reduction from 39.79% to 39.05% in the coil structure of CAT and increments of 0.53% and 0.23% in the turn and sheet structures, respectively. This change suggests that the flexible coil regions transition to more rigid sheet and turn structures and potentially reduce the structural fluctuations and enhance the stability of CAT. These findings from the MD simulations complement the experimental results and provide a molecular‐level understanding on how His_6_ can modulate the structural dynamics of CAT and potentially lead to enhanced catalytic activity and stability.

**Figure 5:**
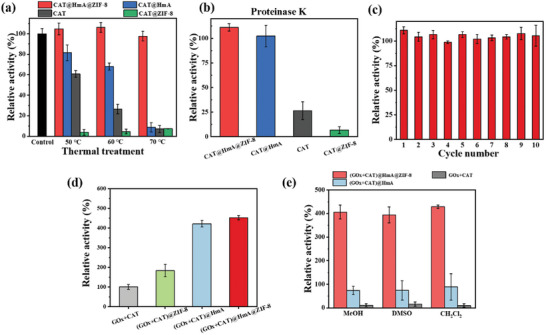
a) High‐temperature and b) proteinase‐K tolerance tests of CAT@HmA@ZIF‐8, CAT@HmA, CAT, and CAT@ZIF‐8. c) Relative enzyme activity after repeated freezing and thawing of CAT@HmA@ZIF‐8 for 10 cycles. d) Cascade enzymatic activities of GOx+CAT, (GOx+CAT)@ZIF‐8, (GOx+CAT)@HmA, and (GOx+CAT)@HmA@ZIF‐8. e) Cascade enzymatic activities of GOx+CAT, (GOx+CAT)@HmA, and (GOx+CAT)@HmA@ZIF‐8 after treatment with organic solvents.

To further illustrate the benefits of the core–shell strategy for resisting external heat, CAT@HmA@ZIF‐8 was dispersed in water at a controlled temperature for 15 min. When heated to 50 and 60 °C, CAT activity decreased significantly to 60.9% and 26.6%, respectively. CAT@HmA exhibited moderate activity at these temperatures, with 81.5% and 68.0% retention, respectively (**Figure** **5**a). CAT@HmA@ZIF‐8 exhibited the highest activity retention (>97%). At 70 °C, the activities of CAT and CAT@HmA decreased to less than 8%, whereas CAT@HmA@ZIF‐8 still retained 97.5% of its activity, indicating an exceptional thermal stability (Figure 5a). CAT@HmA@ZIF‐8 was treated with solutions having different pH values for 30 min to evaluate its tolerance. As shown in Figure S10 (Supporting Information), the catalytic activity of CAT@HmA@ZIF‐8 decreases to 18.3% when exposed to a solution having a pH of 3. However, after incubation in a strongly alkaline solution (pH = 11), CAT@HmA@ZIF‐8 retains 89.6% of its initial activity. Notably, when CAT@HmA@ZIF‐8 is treated with solutions having pH values of 5–9, its activity becomes >108.0%, indicating its exceptional stability and catalytic performance within this pH range. Furthermore, after treatment with proteinase K, CAT activity is only 26.6% of its original level. The catalytic activity of CAT@HmA@ZIF‐8 is 111.1% higher than that of CAT@HmA (102.2% activity), indicating that encapsulation offers protection against enzymatic degradation (Figure 5b). Because of the high stability of CAT@HmA@ZIF‐8, its recyclability was investigated. The enzymatic activity of CAT@HmA@ZIF‐8 did not significantly decrease after 10 cycles (Figure 5c), indicating that the catalyst can be reused effectively.

To explore the versatility of the core–shell strategy, a classic model of cascade enzymes (GOx+CAT) was selected and co‐embedded within HmA@ZIF‐8.^[^ ^60^, ^61^ ^]^As shown in Figure 5d, the cascade activities of GOx and CAT within HmA@ZIF‐8 are 4.5 times higher than that of GOx+CAT, whereas the cascade activity of (GOx+CAT)@ZIF‐8 is only 1.8 times higher than that of GOx+CAT. The improvement in activity may be attributed to the short distance between the two enzymes, resulting in rapid transfer of the intermediate to the second enzyme and high local substrate concentrations.^[^ ^62^ ^]^ Furthermore, the resistance to organic solvents is observed when the samples are treated with MeOH, DMSO, and CH_2_Cl_2_ for 10 min at room temperature. As displayed in Figure 5e, the cascade activity of (GOx+CAT)@HmA@ZIF‐8 remains at approximately 400%, indicating that the core of (GOx+CAT)@HmA with the ZIF‐8 shell provides outstanding protection to enzymes in organic solvents. However, the activities of the other samples decrease distinctly, with approximately 75% for (GOx+CAT)@HmA and 10% for (GOx+CAT), after treatment with organic solvents. These data confirm that the core–shell strategy is an effective approach for enhancing the catalytic activity of diverse enzymes and providing protection from harsh conditions. The distinctive architecture and enzyme‐friendly microenvironment of the core–shell strategy not only enhances catalytic activity but also provides a stable platform for enzyme immobilization, with potential applications in various industries where stability and recyclability are of paramount importance.

**Figure 6:**
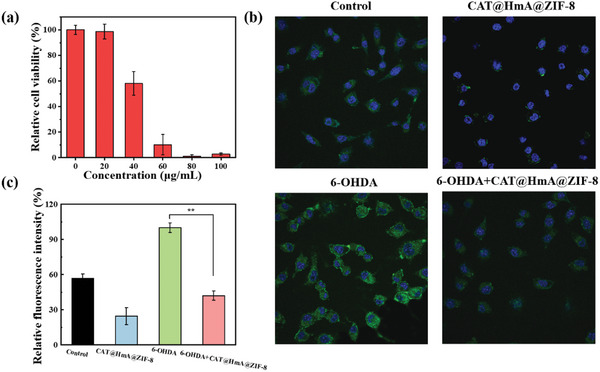
a) Cytotoxicity of CAT@HmA@ZIF‐8 in SH‐SY5Y. b) Confocal fluorescence images of SH‐SY5Y cells stained with 2′,7′‐dichlorodihydrofluorescein diacetate and 4′,6‐diamidino‐2‐phenylindole after different treatments. c) Quantitative analysis of relative green fluorescence intensity measured from confocal fluorescence images.

CAT is essential for the elimination of H_2_O_2_ from the body and prevention of cellular oxidative damage. The biocompatibility and oxidation resistance of CAT@HmA@ZIF‐8 were evaluated in vitro using SH‐SY5Y cells. The relative cell activity was found to be concentration‐dependent when treated with different concentrations of CAT@HmA@ZIF‐8 (**Figure** **6**a). At the CAT@HmA@ZIF‐8 concentration of 20 µg mL^−1^, the survival rate of cells was 98.7%. CLSM was used to assess the antioxidant activity of CAT@HmA@ZIF‐8 (Figure 6b). The control and CAT@HmA@ZIF‐8 groups showed weak green fluorescence, indicating low levels of reactive oxygen species (ROS). By contrast, the group treated with 6‐hydroxydopamine (6‐OHDA), a neurotoxin that increases intracellular ROS levels, exhibited a strong green fluorescence. When CAT@HmA@ZIF‐8 was added to the cells treated with 6‐OHDA, the green fluorescence was significantly reduced, indicating a decrease in ROS levels. The relative green fluorescence intensities were quantified using the ImageJ software (Figure 6c). The fluorescence intensity in the 6‐OHDA + CAT@HmA@ZIF‐8 group was 0.42 times that in the 6‐OHDA group and even lower than that in the control group. This suggests that CAT@HmA@ZIF‐8 is capable of scavenging intracellular ROS and protecting cells from oxidative stress damage. These results highlight that CAT@HmA@ZIF‐8 has potential applications in therapeutic strategies aimed at reducing oxidative stress and protecting cells from the damaging effects of ROS. The ability of an immobilized enzyme to maintain its activity in a biocompatible manner and resist oxidation is a significant advantage in various biomedical contexts.

## 3 Conclusion

We developed a core–shell strategy suitable for the immobilization of a wide range of enzymes (CAT, GOx, HRP, and cascade benzylase (GOx+CAT)) and the preservation of enzyme activities and resistance to external perturbations. The presence of an HmA resulted in an enzyme‐friendly microenvironment that preserved the activities of the encapsulated enzymes. In addition, the shell biomineralized onto enzyme@HmA was robust to resist the disturbances resulting from heat (70 °C) and organic solvents (MeOH, DMSO, and CH_2_Cl_2_). The catalytic activity of the encapsulated enzymes did not decrease even after dozens of cycles of catalysis. Given the mild preparation conditions and straightforward fabrication process required for enzyme@HmA and the versatility and broad applicability of this core–shell strategy, we posit that this strategy marks a significant advancement in the field of enzyme immobilization. This strategy enhances the stability and activity of enzymes and has the potential to expand the range of applications of enzymes in various fields, such as industries, enzyme‐based devices, and biomedicine.

## Conflict of Interest

The authors declare no conflict of interest.

## Author Contributions

Y.W., and H.T. contributed equally to this work. Y.W., H.T., and X.Z. designed the study. Y.W. and S.N. performed most of the experiments. Y.W. and X.Y. analyzed the data and software applications. H.T., K.H., and W.L. contributed intellectually to the study. X.Y. wrote the original manuscript. S.W., X.Z., and X.Y. revised the manuscript. S.W. and X.Z. supervised the study and acquired funding. All authors have read and approved the final manuscript for submission.

## Supporting information



Supporting Information

## Data Availability

The data that support the findings of this study are available from the corresponding author upon reasonable request.
