# Supplementary material for: Combining Hard Shell with Soft Core to Enhance Enzyme Activity and Resist External Disturbances
Source: Adv Sci (Weinh). 2025 Jan 22;12(10):2411196. doi: 10.1002/advs.202411196 (PMC11905098; doi:10.1002/advs.202411196)
Supplement: Supplementary file 1 — Supporting Information [file ADVS-12-2411196-s001.docx]

**Supporting Information**

Combining Hard Shell with Soft Core to Enhance Enzyme Activity and Resist External Disturbances

*Yiwen Wang,*^§,^ *^♀, #^ Hongfei Tong, ^¶, #^ Shulan Ni,*^§,^ *^♀^ Kaiyuan Huo,*^§,^ *^♀^ Wenjie Liu,^♀, Ѱ^ Xingj**ie Zan, ^♀^ * Xiaodie Yuan,**^♀^ * and Shuangshuang Wang*^§^ *^Ʈ^*^*^

^§^ Department of Cardiology, The First People’s Hospital of Wenling, Wenling Hospital of Wenzhou Medical University, Wenling 317500, Zhejiang Province, China

*^♀^* Wenzhou Institute, University of Chinese Academy of Sciences, Wenzhou Key Laboratory of Perioperative Medicine, Wenzhou, Zhejiang, 325001, China

*^¶^* Yongkang First People's Hospital of Wenzhou Medical University, Jinhua, Zhejiang Province, China.

*^Ѱ^* School of Materials Science and Engineering, Zhengzhou University, Zhengzhou 450001, China

*^Ʈ^* Key Laboratory of Precision Medicine for Atherosclerotic Diseases of Zhejiang Province, Affiliated First Hospital of Ningbo University, Ningbo 315010, Zhejiang Province, China

*^#^* Equal contribution author

*Corresponding author. E-mail: [wangss1023@126.com](mailto:wangss1023@126.com) (S. Wang); zanxj@ucas.ac.cn (X. Zan); yuanxiaodie@ucas.ac.cn (X. Yuan);

**Experimental Methods**

**Materials**

2-Methylimidazole (2-MI), DCFH-DA and 2-imidazolate carboxaldehyde (ICA) were purchased from Shanghai Macklin Biochemical Technology Co., Ltd. Zn(CH_3_COO)_2_•2H_2_O, Na_2_HPO_4_, citric acid and 6-HydroxyDopamineHydrochloride (6-OHDA) were obtained from Shanghai Aladdin Biochemical Technology Co., Ltd. Catalase (CAT) and Horseradish peroxidase (HRP) were bought from Shanghai yuanye Bio-Technology Co., Ltd. Glucose oxidase (GOx) was obtained from Beyotime Biotech Inc. Hexahistidine (His_6_) and FITC-His6 were bought from Nanjing TGpeptide Biotechnology Co.,Ltd. Proteinase K was acquired from Beijing Worbisen Technology Co., Ltd. DMEM was provided by Gibco Life Technologies. CCK-8 was obtained from Vazyme Biotech Co.,Ltd.

**Characterization.** Scanning electron microscopy images were obtained on a Hitachi SU8010 Field-Emission Scanning Electron Microscope. UV-vis spectra were record on a Cary 5000 UV-Vis-NIR spectrophotometer. The encapsulating contents of CAT were measure by a UV-Vis-NIR spectrophotometer and a 1260 Infinity II HPLC system in MeCN/H_2_O (v : v = 8 : 2 ) at a flow rate of 0.4 mL min^−1^ and detection wavelength of 240 nm, respectively. The XRD patterns were acquired on a D8 Advance. The fluorescence images were performed with a Nikon A1 confocal laser-scanning microscope. The hydrodynamic sizes and zeta potentials were recorded with a Malvern Zetasizer Nano ZS ZEN3600 apparatus. The surface area and pore size were tested by nitrogen adsorption isotherms with a Micromeritics 3 Flex instrument. FT-IR spectra were conducted on a Tensor II spectrometer.

**Preparation of Enzyme@HmA, Enzyme@HmA@ZIF-8, Enzyme@****ZIF-8/ZIF-90/ZIF-67/CaCO_3_/HAP and Enzyme@HmA@****ZIF-8/ZIF-90/ZIF-67/CaCO_3_/HAP particles**. For Enzyme@HmA particles, 2 mg His_6_ and 2 mg Enzyme (GOx, HRP, CAT) were dissolved in 500 μL HEPES-Na buffer solutions (pH=8.5). Then, Zn(NO_3_)_2_ ( 24 μL, 0.1 M )solution was added and ultrasonic for 15 min. Finally, the sample was collected by centrifuge at 8000 rpm/min, and washed three times with water. For Enzyme@ZIF-8 particles, 2 mg Enzyme was dissolved in 2-MI solution (2 mL, 0.16 M). Then, triethylamine (30 μL) was added with stirring at 500 rpm for 1 min. Then, Zn(CH_3_COO)_2_ (2 mL, 0.04 M) solution was poured into it and stirred at 500 rpm for 15 min. And after standing for 8 h, the Enzyme@ZIF-8 particles were collected by centrifugation at 5000 rpm and washed for three times. For Enzyme@ZIF-67 particles, Enzyme (2 mg) was dissolved in Co(NO_3_)_3_ solution (2 mL, 0.05 M), and stirring for 1 min at 500 rpm. Then, 2-MI (2 mL, 0.5 M) solution was poured into it and stirred for 30 min. And after standing for overnight, the Enzyme@ZIF-67 particles were collected by centrifugation at 5000 rpm and washed for three times. For Enzyme@ZIF-90 particles, Enzyme (2 mg) was dissolved in Zn(NO_3_)_2_ solution (2 mL, 37.1 mg/mL), and stirring for 1 min at 500 rpm. Then, imidazole-2-carboxaldehyde solution (1.5 mL, 32 mg/mL) was poured into it and stirred for 30 min. And after standing for overnight, the Enzyme@ZIF-90 particles were collected by centrifugation at 5000 rpm and washed for three times. For Enzyme@CaCO_3_ particles, Enzyme (2 mg) was dissolved in CaCl_2_ solution (2 mL, 0.5 M), and stirring for 1 min at 1200 rpm. Then, Na_2_CO_3_ solution (2 mL, 0.5 M) was poured into it and stirred for 5 min. And after standing for 15 min, the Enzyme@CaCO_3_ particles were collected by centrifugation at 5000 rpm and washed for three times. For Enzyme@HAP particles, Enzyme (2 mg) was dissolved in CaCl_2_ solution (2 mL, 0.1 M), and Na_2_HPO_4_ solution (0.1 M) was dropped into it and adjusted the pH to 8.5. And after stirring for 24 h at 37 ℃, the Enzyme@HAP particles were collected by centrifugation at 5000 rpm and washed for three times. For Enzyme@HmA@ZIF-8/ZIF-90/ZIF-67/CaCO_3_/HAP particles, the enzyme was replaced with the above Enzyme@HmA particles and other steps were the same as the preparation process of Enzyme@porous materials.

**Evaluation of CAT catalytic activity.** The catalytic activity of CAT was detected by FOX assay. In the presence of H_2_O_2_, the color of FOX solution (including 25 mM H_2_SO_4_, 100 mM sorbitol, 100 μM xylenol orange and 250 μM ammonium ferrous sulfate) changed from lemon yellow to purple. The H_2_O_2_ concentration was measured by mixing FOX solution, H_2_O_2_ and nanoparticles. In brief, firstly H_2_O_2_ solution (500 μL, 150 μM) was mixed with 500 μL of nanoparticles solution for 120s. Then, 50 μL of above reaction solution was added into the 950 μL of FOX solution and incubating for 30 min in the dark. Finally, the absorbance at 570 nm was recorded with UV-vis spectrometer.

**Measurement of CAT encapsulating capacity in CAT@HmA and CAT@HmA@ZIF-8.** The CAT loading contents were tested by HPLC and UV-vis. The standard curve of CAT concentration versus area at 240 nm was established. In the typical experiment, 10 mg of nanoparticles was dissolved in the pH 4.0 solution to release CAT. Then the amount of CAT in nanoparticles was determined by the HPLC and UV-vis.

**Evaluation of enzyme stability.** To evaluate the enzyme stability after thermal and solvent treatments, the Enzyme, Enzyme@HmA, Enzyme@ZIF-8/ZIF-90/ZIF-67/CaCO_3_/HAP and Enzyme@HmA@ZIF-8/ZIF-90/ZIF-67/CaCO_3_/HAP were incubated in 50/60/70 ℃ water for 15 min and organic solvent solution (MeOH, DMSO and CH_2_Cl_2_) for 10 min, respectively.

The Proteinase K tolerance of CAT, CAT@ZIF-8, CAT@HmA and CAT@HmA@ZIF-8 was tested by incubating in 2 mg/mL Proteinase K solutions for 20 min at 37 °C. The CAT activities were measured with the above-mentioned method.

**Measurement of CAT@HmA@ZIF-8 freezing and thawing recyclability.** The recyclability of CAT@HmA@ZIF-8 was measured for 10 cycles. For each cycle, the CAT@HmA@ZIF-8 nanoparticles solutions were frozen and thawed, and then collecting by centrifugation and the samples were added in the FOX solutions. After that, the nanoparticles were washed five times with PBS, and reused in the next cycle.

**Measurement of the kinetic parameters.** The enzymatic kinetic of CAT and CAT doped nanoparticles were tested by monitoring the absorbance variation at 570 nm over time after mixing sample solutions with FOX solutions. The kinetic parameters were calculated by the Michaelis-Menten equation (1).

$V=\frac{V_{max}[S]}{Km+[S]}$(1)

$\frac{1}{V}=\frac{Km}{V_{max}}\times\frac{1}{[S]}+\frac{1}{V_{max}}$(2)

Here, V is the initial rate of enzymatic reaction, V_max_ is the maximum rate of enzymatic reaction which indicates the rate of reaction at equilibrium. [S] is the substrate concentration. K_m_ is the Michaelis constant which represents the binding ability of enzyme and substrate. Then, the Lineweaver–Burk plots (2) are made by taking reciprocal deformation of both sides of the Michaelis-Menten equation. V_max_ and K_m_ can be calculated from the slope and intercept of the Lineweaver–Burk plot.

**Cell Culture.** The SH-sy5y cells were cultured in Dulbecco’s modified eagle medium (DMEM) supplemented with 10% fetal bovine serum and 1% Penicillin-Streptomycin at 37 °C in a humidified atmosphere of 5% CO_2_.

***In vitro* Cytotoxicity Assay of CAT@HmA@ZIF-8.** SH-sy5y cells were seeded into 96-well plates at a density of 5000 cells per well. After culturing for 12 h, the medium was discarded, cleaning with PBS for 3 times. Then, different concentration CAT@HmA@ZIF-8 nanoparticles were added into wells. The cells were subsequently incubated for 24 h in the CO_2_ incubator. After that, 10 μL of CCK8 solution was added to each well to a final volume of 100 μL, and place the 96 well plate into the CO_2_ incubator for 2 h. The absorbance at 450 nm was recorded with a microplate reader. The cell viability was tested according to the following equation: Cell viability (%) = (OD_nanoparticles_/OD_control_) × 100%. Where OD_nanoparticles_ was obtained in the prescence of nanoparticles, and OD_control_ was in the abscence of nanoparticles.

**Determination of CAT@HmA@ZIF-8 ability to capture intracellular ROS.** SH-sy5y cells were seeded on the glass slides at a density of 50000 cells per square centimeter. After incubation for 12 h, the CAT@HmA@ZIF-8 was added into the well as the experimental group. And then the cells treated with or without 300μM 6-OHDA for 12 h, which can upregulate intracellular ROS levels. The cells were washed with PBS twice and incubated with 10 μM of DCFH-DA for 30 min at 37 ℃. After the unloaded probe was removed with PBS, the cells were dyed with DAPI and the fluorescence intensity of cells was imaged by fluorescence microscopy.


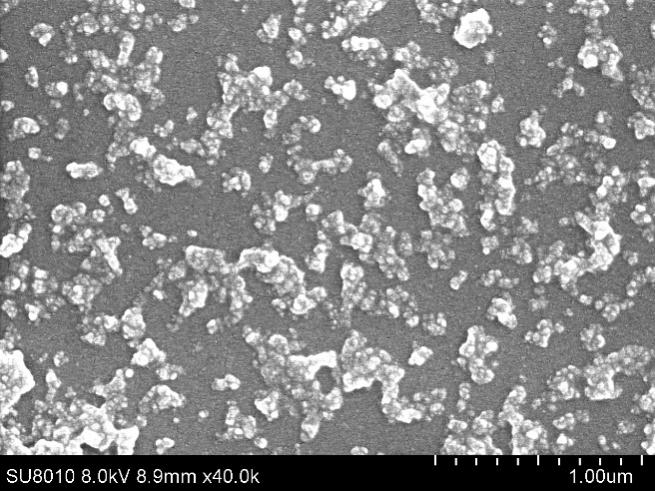


**Figure S1.** The scanning electron microscope image of CAT@HmA.


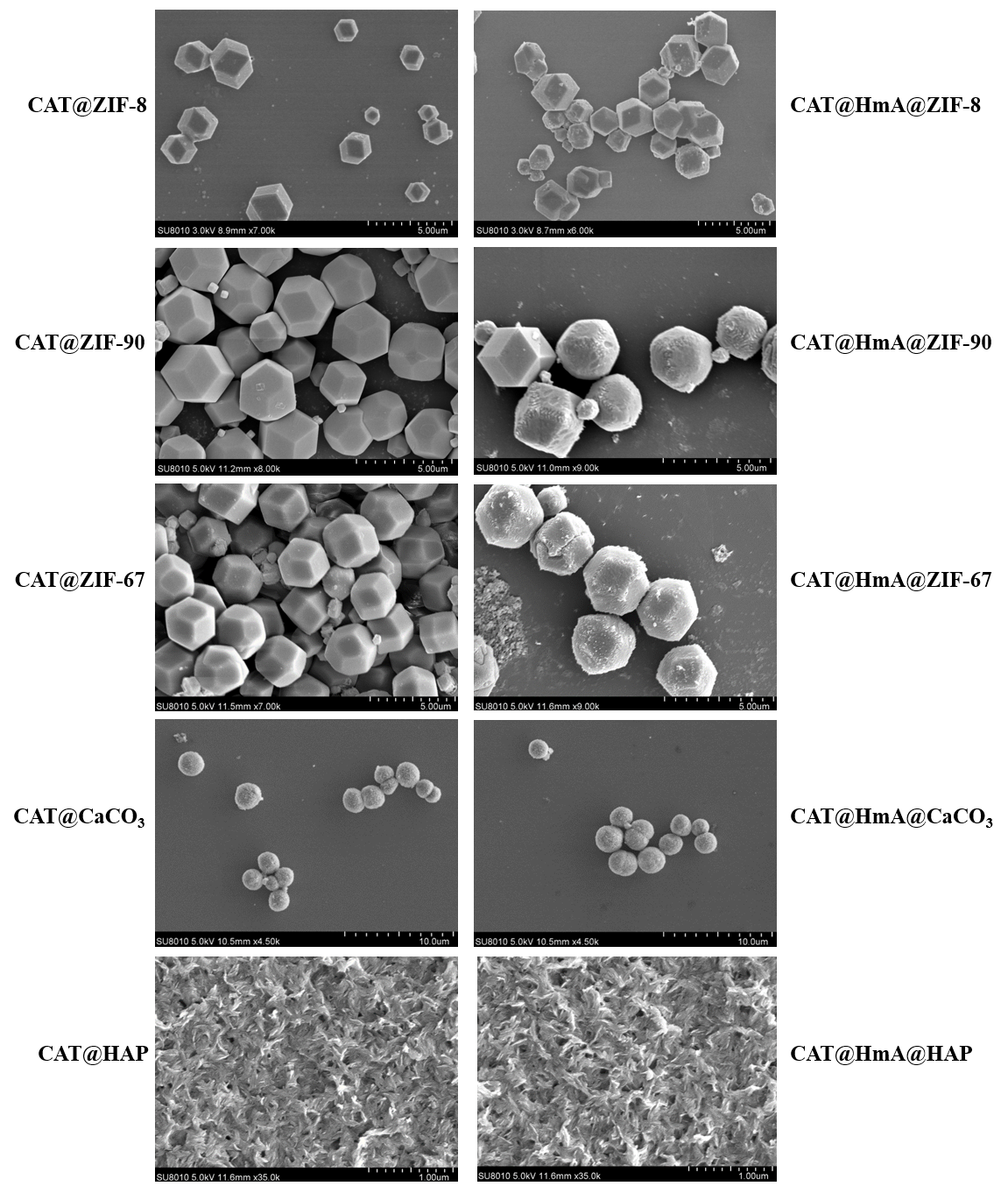


**Figure S2.** The scanning electron microscope images of CAT@ZIF-8/ZIF-90/ZIF-67/CaCO_3_/HAP and CAT@HmA@ZIF-8/ZIF-90/ZIF-67/CaCO_3_/HAP.


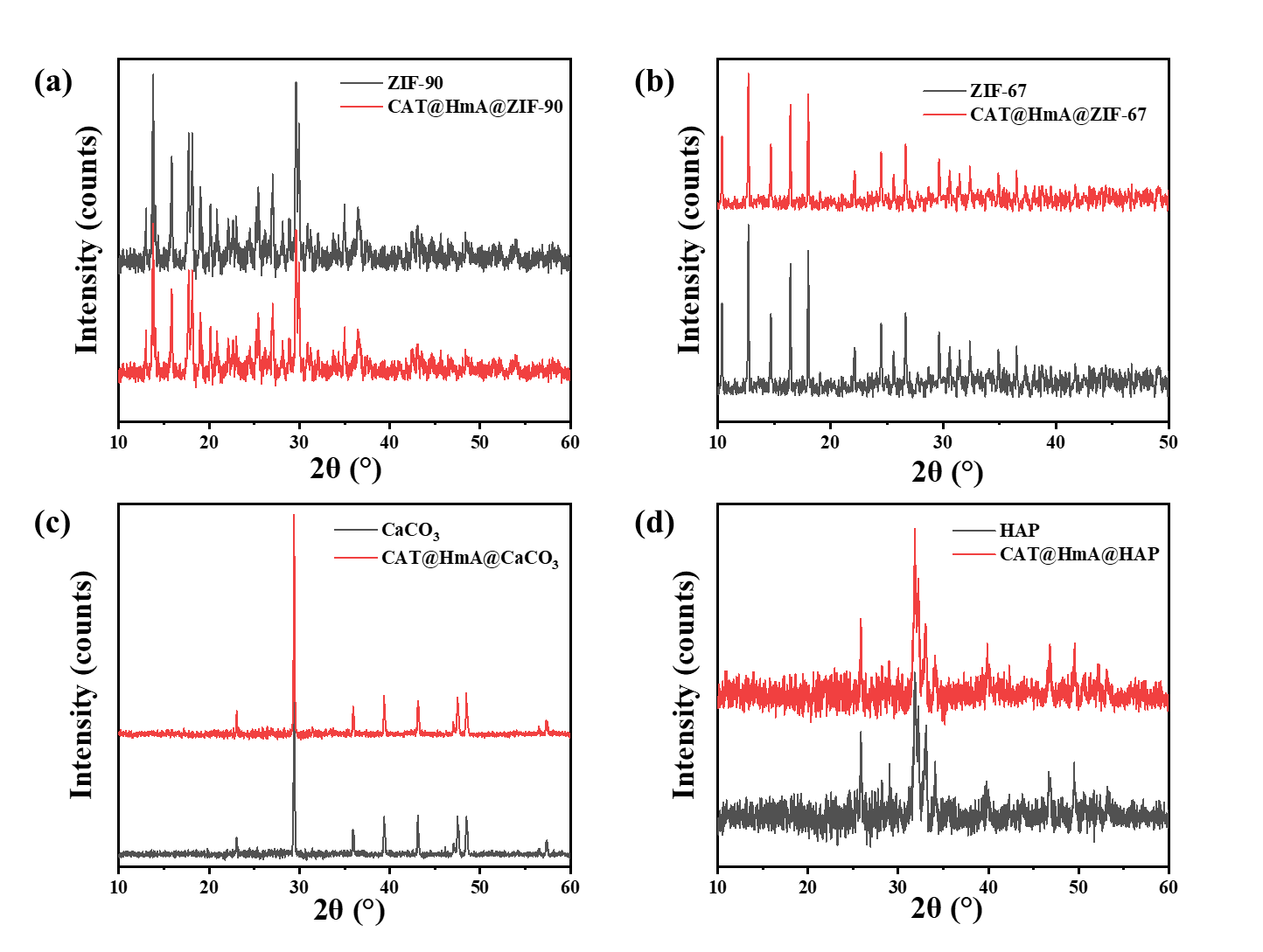


**Figure S3.** XRD patterns of (a) ZIF-90 and CAT@HmA@ZIF-90, (b) ZIF-67 and CAT@HmA@ZIF-67, (c) CaCO_3_ and CAT@HmA@CaCO_3_, (d) HAP and CAT@HmA@HAP.


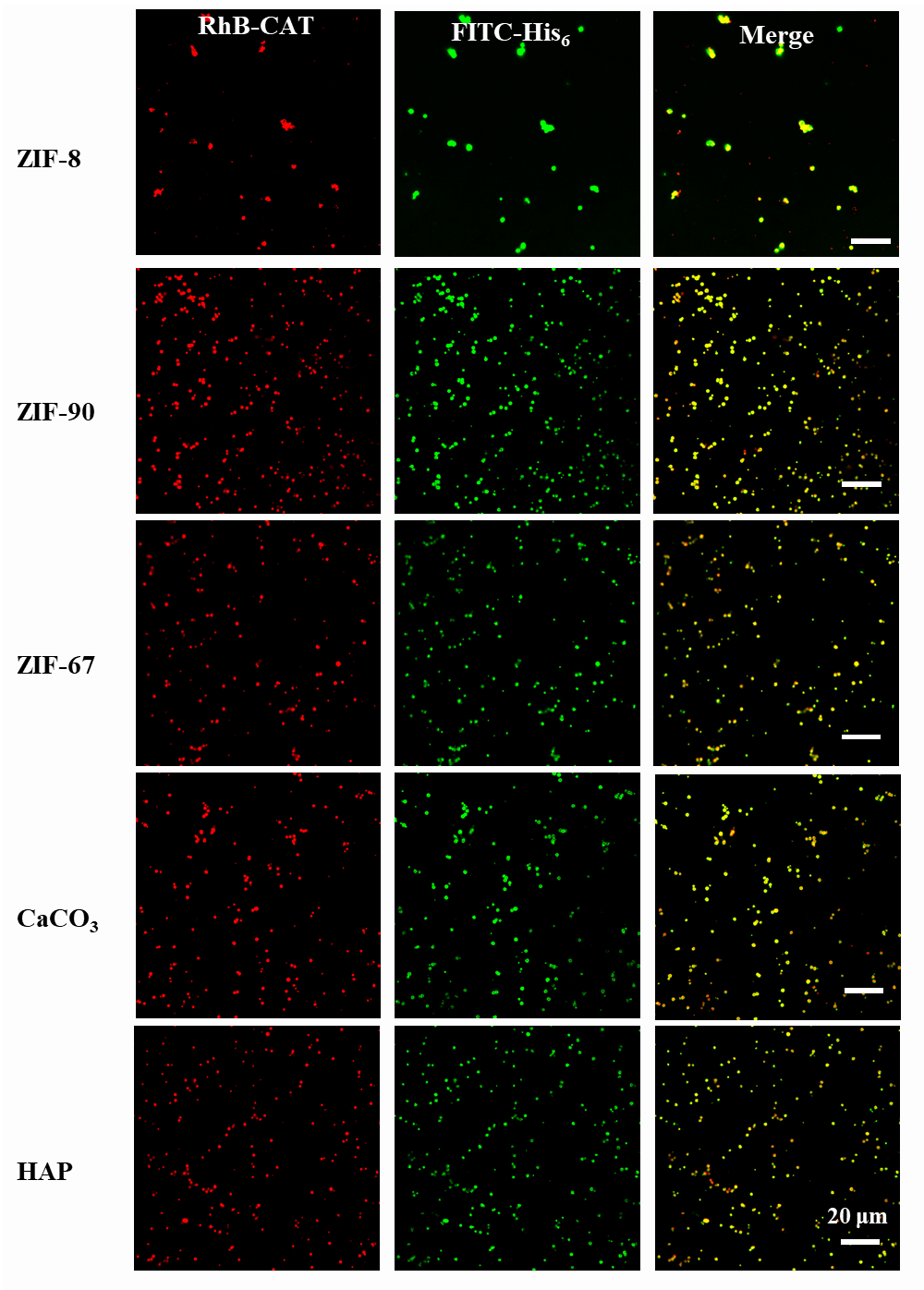


**Figure S4.** CLSM images showed the distribution of His_6_ and CAT within core-shell CAT@HmA@ZIF-8/ZIF-90/ZIF-67/CaCO_3_/HAP. CAT was labelled with Rhodamine B (RhB), and His_6_ was labelled with FITC.


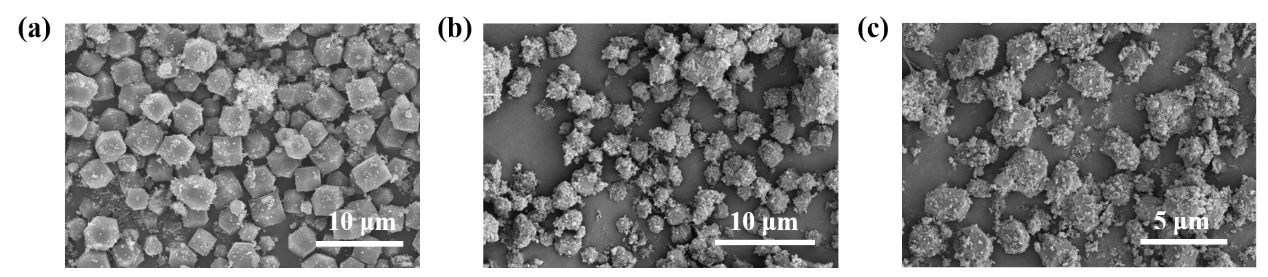


**Figure S5.** The scanning electron microscope images of (a) ZIF-8, (b) CAT@ZIF-8 and (c) CAT@HmA@ZIF-8 after high temperature carbonization treatment.


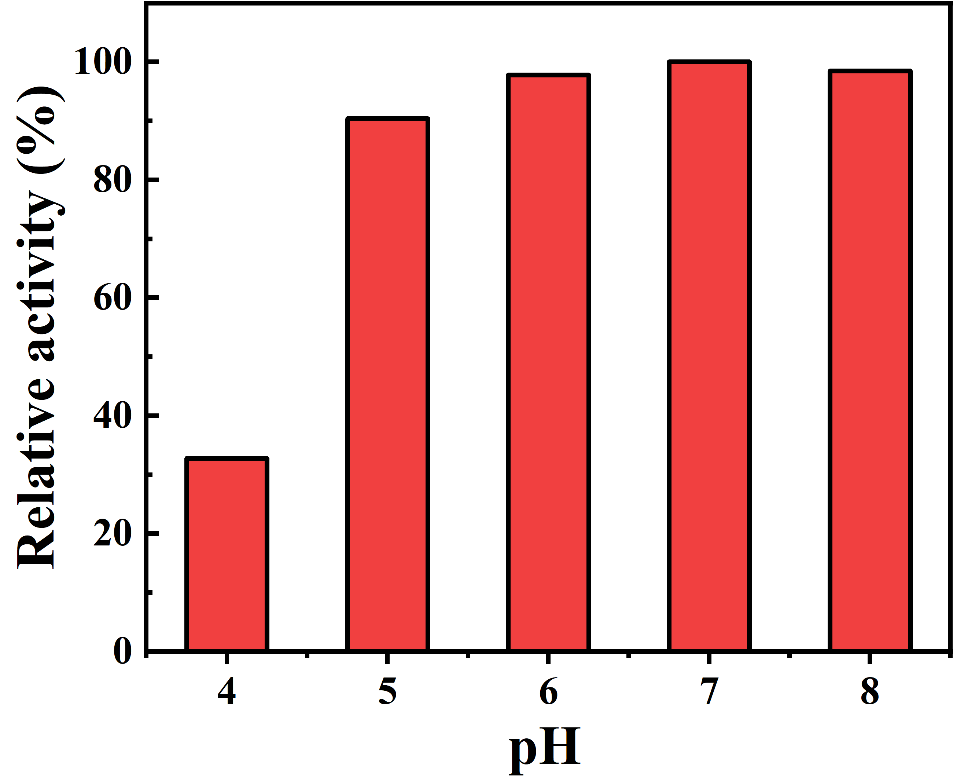


**Figure S6.** Relative activity of CAT under different pH solutions.


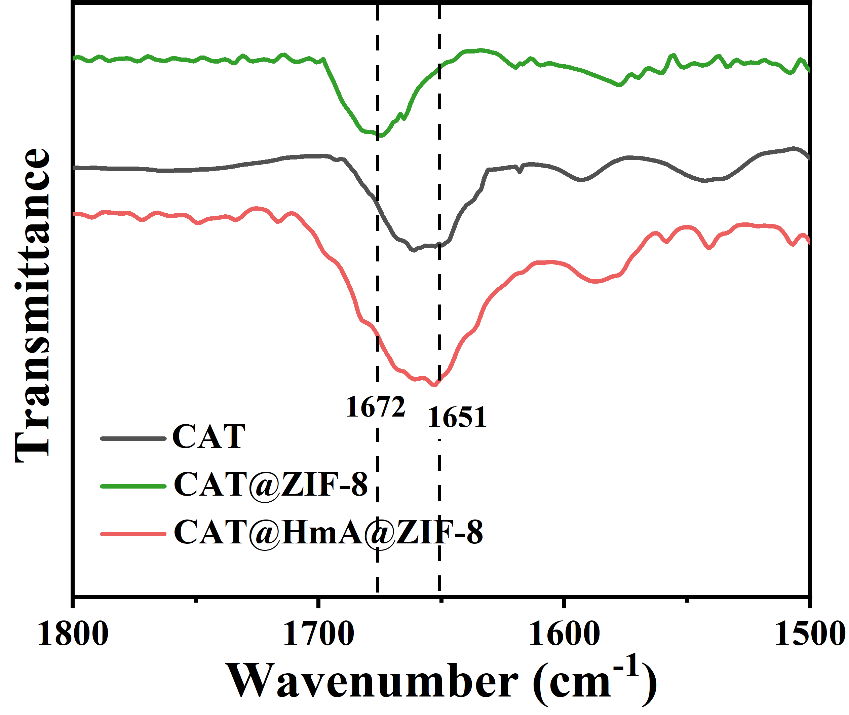


**Figure S7.** FT-IR spectra of CAT, CAT@ZIF-8 and CAT@HmA@ZIF-8.


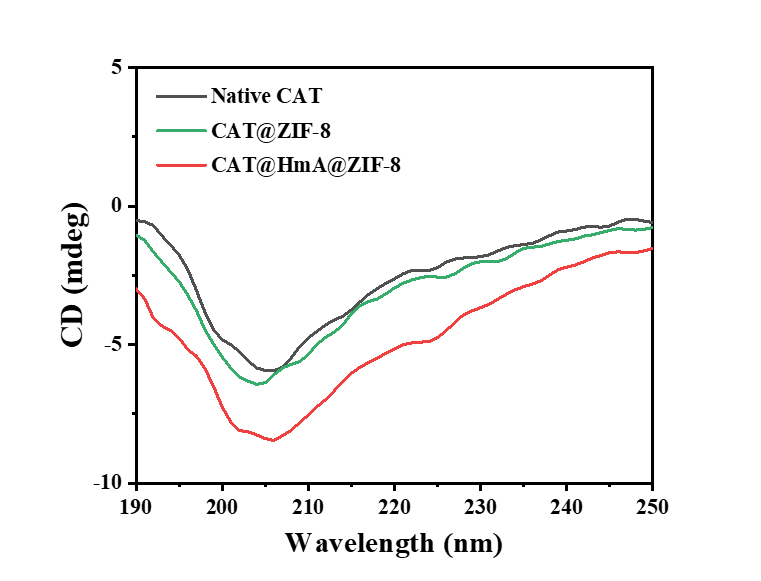


**Figure S8.** CD spectra of native CAT, CAT@ZIF-8 and CAT@HmA@ZIF-8.


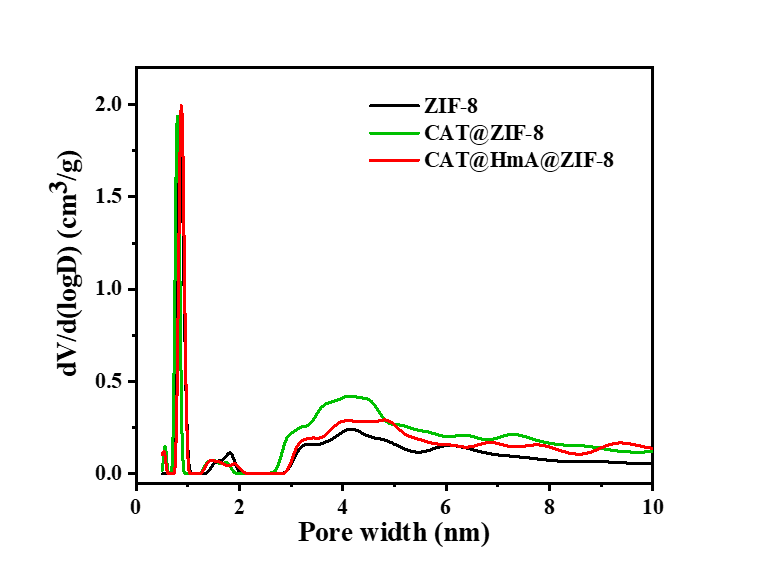


**Figure S9.** Average aperture curve of CAT@HmA@ZIF-8, ZIF-8 and CAT@ZIF-8.


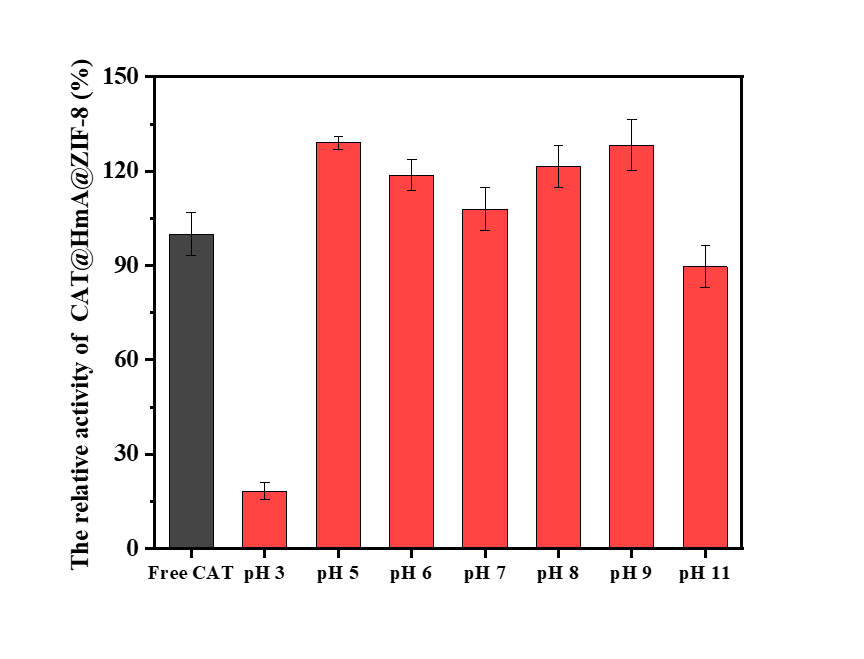


**Figure S10.** The relative activity of CAT@HmA@ZIF-8 in different pH solutions.

**Table S1.** The hydrodynamic diameters, Polymer dispersity index (PDI), zeta potentials and encapsulation efficiency (EE%) of CAT@HmA, CAT@ZIF-8, CAT@HmA@ZIF-8, CAT@HmA@ZIF-90, CAT@HmA@ZIF-67, CAT@HmA@CaCO_3_ and CAT@HmA@HAP.

|  | CAT@HmA | CAT@ZIF-8 | CAT@HmA@ZIF-8 | CAT@HmA@ZIF-90 | CAT@HmA@ZIF-67 | CAT@HmA@CaCO_3_ | CAT@HmA@HAP |
| --- | --- | --- | --- | --- | --- | --- | --- |
| Size (nm) | 213.6±18.4 | 1537.1±341.9 | 1392.7±248.9 | 1912.4±218.8 | 2361.1±312.9 | 3621.0±249.1 | 519.6±107.1 |
| PDI | 0.17 | 0.25 | 0.22 | 0.24 | 0.34 | 0.26 | 0.37 |
| Zeta (mV) | 18.2±2.1 | 22.6±1.8 | 21.4±2.0 | -13.6±1.6 | 16.4±1.4 | 19.6±1.2 | -12.6±1.1 |
| EE% | 98±6 | 92±4 | 91±3 | 90±4 | 93±5 | 88±2 | 88±3 |

**Table S2.** Relative activities of free enzyme, enzyme@HmA (core), enzyme@shell (enzyme directly immobilized on the shell), and enzyme@HmA@shell (enzyme immobilized by core-shell strategy). The enzymes included GOx, HRP, and CAT; the shell comprised ZIF-8, ZIF-90, ZIF-67, CaCO_3_, and HAP. The data were normalized against the activity of free enzymes at room temperature.

| **Relative activity %** | | **GOx** | **HRP** | **CAT** |
| --- | --- | --- | --- | --- |
|  |  |  |  |  |
| **Free enzyme** |  | 100.0±2.4 | 100.0±3.5 | 100.0±3.2 |
|  | **@HmA** | 106.1±7.2 | 94.8±4.2 | 105.6±3.2 |
| **ZIF-8** |  | 143.7±4.3 | 176.8±3.2 | 8.1±4.2 |
|  | **@HmA** | 151.9±7.2 | 169.5±10.8 | 111.1±5.5 |
| **ZIF-67** |  | 24.9±11.3 | 87.5±6.9 | 21.1±10.3 |
|  | **@HmA** | 84.7±5.2 | 82.9±14.7 | 89.9±6.2 |
| **ZIF-90** |  | 107.3±6.0 | 12.7±4.2 | 69.3±7.3 |
|  | **@HmA** | 97.2±12.2 | 85.1±3.6 | 94.3±7.2 |
| **CaCO_3_** |  | 89.4±7.1 | 81.0±8.5 | 87.8±7.3 |
|  | **@HmA** | 88.1±3.1 | 91.5±3.7 | 98.1±2.6 |
| **HAP** |  | 91.1±11.4 | 89.6±7.6 | 96.3±1.8 |
|  | **@HmA** | 73.1±4.3 | 104.0±8.5 | 98.7±3.2 |

**Note.** When the catalytic activity of the enzyme@shell significantly decreased, the corresponding area of the enzyme@shell was filled with gray. Then, define the value of A as the catalytic activity of aforementioned enzyme@HmA@shell to enzyme@shell. Values of A within the range 1-3 are represented in blue, those between 3.1 and 9.9 in yellow, and values exceeding 10 in red.

**Table S3.** Comparison of kinetic parameters for free CAT, CAT@HmA, CAT@HmA@ZIF-8 and CAT@ZIF-8.

|  | CAT | CAT@HmA | CAT@HmA@ZIF-8 | CAT@ZIF-8 |
| --- | --- | --- | --- | --- |
| V_max_ (μM/s) | 144.9 | 169.4 | 153.8 | 1.4 |
| Km (μM) | 133.0 | 148.9 | 111.0 | 4.6 |

**Table S4.** The specific surface areas of ZIF-8, CAT@ZIF-8 and CAT@HmA@ZIF-8.

|  | Specific surface area (m²/g) |
| --- | --- |
| ZIF-8 | 629.7 |
| CAT@HmA@ZIF-8 | 663.1 |
| CAT@ZIF-8 | 656.0 |
